# A randomised controlled trial of Acceptance and Commitment Therapy plus usual care in comparison to usual care alone for reducing anxiety in older people with treatment-resistant generalised anxiety disorder (CONTACT-GAD): trial protocol

**DOI:** 10.1186/s12877-026-07094-6

**Published:** 2026-02-11

**Authors:** Rebecca L. Gould, Tia Callaghan, David White, Julie Loebach Wetherell, Matt Bursnall, Mike Bradburn, Allan Wailoo, Marc A. Serfaty, Christopher D. Graham, Robert J. Howard, Philip Wilkinson, Lucy Musson, David Ekers, Kate Walters, Gill Livingston, Viviana M. Wuthrich

**Affiliations:** 1 Division of Psychiatry, University College London, Wing B, 4th floor Maple House, 149 Tottenham Court Rd, London, W1T 7NF UK; 2 Clinical Trials Research Unit, School of Health and Related Research, University of Sheffield, Sheffield, UK; 3 Mental Health Service, VA San Diego Healthcare System, San Diego, CA USA; 4 Department of Psychiatry, University of California San Diego, San Diego, CA USA; 5 School of Health and Related Research, University of Sheffield, Sheffield, UK; 6 Priory Hospital North London, London, UK; 7 Department of Psychological Sciences & Health, University of Strathclyde, Glasgow, UK; 8 Department of Psychiatry, University of Oxford, Oxford, UK; 9 Sheffield Institute for Translational Neuroscience, University of Sheffield, Sheffield, UK; 10 Health Sciences, University of York, York, UK; 11 Department of Primary Care and Population Health, University College London, London, UK; 12 Lifespan Health & Wellbeing Research Centre, Macquarie University, Sydney, Australia

**Keywords:** Older people, Generalised anxiety disorder, Treatment resistant, Acceptance and Commitment Therapy, RCT

## Abstract

**Background:**

Generalised anxiety disorder (GAD) is the most common anxiety disorder in older people and is characterised by excessive anxiety and worry that is experienced as being difficult to control. Current recommended first-line treatments for GAD include pharmacotherapy and psychological therapy, but some people experience GAD that does not respond to these treatments. Such treatment-resistant GAD (TR-GAD) is associated with numerous negative outcomes in older people. However, evidence-based guidance on how to manage TR-GAD in older people is lacking. Previous research suggests that Acceptance and Commitment Therapy (ACT), tailored to the needs and preferences of older people with TR-GAD, may help reduce anxiety in this population.

**Aims:**

To determine the clinical and cost-effectiveness of tailored ACT plus usual care (UC) in comparison to UC alone for reducing anxiety in older people with TR-GAD.

**Methods:**

The CONTACT-GAD trial is an international, multi-centre, parallel, two-arm RCT with a 9-month internal pilot phase. 296 individuals aged ≥ 60 years with TR-GAD will be recruited from primary and secondary care services (and their equivalent in Australia) and via self-referral at approximately 11 UK sites and 4 Australian sites. TR-GAD will be defined as GAD that has failed to respond adequately to pharmacotherapy and/or psychotherapy, as described in step 3 of the UK's stepped care model for GAD (and its equivalent in Australia). Participants will be randomly allocated to receive up to 14 one-to-one sessions of ACT with a booster session at approximately 3-months post-intervention plus UC or UC alone by an online randomisation system. Participants will complete outcome measures at baseline and 6- and 12-months post-randomisation. The primary outcome will be anxiety at six months. Secondary outcomes will include quality of life, depression, psychological flexibility, resource use, health-related quality of life, capability, adverse events, satisfaction with therapy, personally meaningful behaviour change and engagement in activities. Outcome assessors will be blind to treatment allocation. Primary analyses will be by intention-to-treat, with data being analysed using multi-level modelling.

**Discussion:**

The CONTACT-GAD trial will provide much needed evidence on the management of TR-GAD in older people.

**Trial registration:**

ISRCTN Registry, https://www.isrctn.com/ISRCTN85462326, registered 04/01/2023.

**Protocol version:**

3.0 (09/05/2025).

**Supplementary Information:**

The online version contains supplementary material available at 10.1186/s12877-026-07094-6.

## Introduction

### Background and rationale

Generalised anxiety disorder (GAD) is the most frequently occurring anxiety disorder in later life, with prevalence rates of up to 11% being observed in this population [1]. It is characterised by excessive worry and anxiety, experienced as being difficult to control, and is accompanied by a range of symptoms, including irritability, fatigue and a sense of dread or unease [2]. It frequently persists for many years and is linked to numerous negative outcomes, including poorer quality of life, and increased disability and use of healthcare services [3]. It is frequently comorbid with other psychiatric disorders, including depression and other anxiety disorders, which further exacerbate negative outcomes [4].

Current clinical guidance recommends a stepped-care approach to the management of GAD within the UK [5]. This ranges from: a) identification and assessment in Step 1; b) low-intensity psychological interventions such as guided cognitive behavioural therapy (CBT) self-help in Step 2; c) pharmacotherapy (such as selective serotonin reuptake inhibitors) and/or high-intensity, psychological interventions (either CBT or applied relaxation) in Step 3; and d) referral to specialist mental health services in Step 4, where treatment options include a combination of treatments from previous Steps. However, it has been estimated that up to 40% of people experience anxiety disorders, including GAD, that do not respond to such first-line treatments [6]. Unfortunately, evidence-based guidance on the management of treatment-resistant GAD (TR-GAD) in older people is lacking due to the limited studies in this area [7]. This prompted the National Institute for Health and Care Research (NIHR) to issue a commissioned call for a randomised controlled trial (RCT) to evaluate the clinical and cost-effectiveness of a psychological intervention for older people with TR-GAD. This protocol describes an RCT that was developed in response to this commissioned call [8].

A form of psychological therapy that may be particularly suitable for older people with TR-GAD is Acceptance and Commitment Therapy (ACT) [9]. ACT is an acceptance-based behavioural therapy with an evidence base in a range of mental and physical health conditions relevant to older people with TR-GAD, including anxiety, depression and chronic pain [10]. It differs from other psychological therapies, such as conventional CBT and applied relaxation, as it is focused on increasing personally meaningful behaviour in the presence of distress and symptoms, rather than being focused on symptomatic reduction. Although ACT and ACT-based approaches have been shown to be as effective as conventional CBT and applied relaxation for GAD in working age adults [11–13], less is known about its effectiveness in older people with GAD [14, 15]. A small, preliminary RCT showed that ACT may be as beneficial as CBT in older people with GAD, but may confer additional benefits with respect to treatment completion [16]. A cluster RCT of older people aged 55–75 years with mild to moderately severe anxiety symptoms reported that blended ACT was as clinically and cost effective as CBT [17, 18]. However, these studies did not specifically examine TR-GAD in older people and so whether ACT is effective for this population is unknown.

In the only study, to the authors’ knowledge, to have developed and evaluated a psychological intervention specifically for older people with TR-GAD, we showed that ACT was both feasible and acceptable for this population [19]. In addition, signals of efficacy with respect to reductions in anxiety, depression and psychological inflexibility (which ACT aims to decrease) from baseline to 20 weeks were demonstrated, with a reliable change in anxiety being seen in 45% of participants. However, whether these beneficial effects were due to ACT was unclear since this was an uncontrolled feasibility study. Furthermore, whether this approach is clinically and cost-effective in this population and whether any beneficial gains are maintained beyond 20 weeks remains to be examined. Consequently, we will evaluate the clinical and cost effectiveness of tailored ACT plus usual care (UC) in comparison to UC alone for reducing anxiety in older people with TR-GAD at 6- and 12-months post-randomisation.

### Objectives

The objectives are to:

1. Adapt our previously developed intervention [19] and all study procedures for remote delivery to increase accessibility.
2. Assess the clinical and cost effectiveness of ACT, tailored to the needs of older people with TR-GAD, plus UC compared to UC alone for reducing anxiety in this population in an RCT with a 9-month internal pilot phase.
3. Examine perceived mechanisms of impact, facilitators of and barriers to implementation, and the context in which the intervention is delivered through qualitative and quantitative data from older people with TR-GAD and trial therapists.
4. Make further refinements to the intervention based on qualitative and quantitative findings, particularly with respect to implementation in clinical practice.
5. Engage the public, stakeholders and mental health services to ensure readiness for implementation in clinical practice (if the intervention is found to be effective).

## Methods

This protocol is reported in accordance with SPIRIT guidelines for clinical trial protocols [20] and TIDIER guidelines for reporting of interventions [21]. See Supplementary Files 1–3 for corresponding checklists and trial registration details.

### Design

This will be an international, multi-centre, outcome assessor-blind, parallel, two-arm RCT with a 9-month internal pilot phase to assess the acceptability of randomisation and feasibility of recruitment. The stop/go criteria for progression to the full RCT are listed in Table 1.

**Table 1:** Stop/go criteria for progression to the full RCT

**Progression criteria**	**Red:** < 50%	**Amber:** 50%−99%	**Green:** 100%
1. Trial recruitment % complete	< 17% of total	17–32% of total	33% of total
2. Recruitment rate/site/month	< 0.37/site/month	0.37–0.72/site/month	0.73/site/month
3. No. of sites opened	≤ 6	7–14	15
4. Total no. of participants recruited	< 50	50–98	99
5. Completion of 7/14 sessions	< 50%	50–99%	100%
6. % of sessions rated with a total ACT inconsistency score of < 18 on the ACT Fidelity Measure	< 50%	50–99%	100%

### Setting

Older people with TR-GAD will be recruited from primary care services (e.g., GP practices, NHS Talking Therapies and third sector organisations that receive primary care referrals and provide psychological therapies), secondary care services (e.g., community mental health teams), and via self-referral. Participants in Australia will be recruited from equivalent healthcare settings and providers. Participants will be recruited from approximately 11 UK sites and 4 Australian sites.

### Eligibility criteria

#### Older people with TR-GAD

Inclusion criteria:

1. Aged ≥ 60 years.
2. GAD diagnosis identified using the Mini-International Neuropsychiatric Interview [22].
3. Since there is no universally agreed definition of TR-GAD in older people, it will be defined here as GAD that has failed to respond adequately (i.e., continued symptoms of GAD that are still causing difficulties) to pharmacotherapy and/or psychotherapy treatment, as described in step 3 of the UK’s stepped-care model for GAD [5]. Those who have been offered treatment and did not want to start it or continue it and are still symptomatic will also be included in this definition. An equivalent definition will be used in Australia. If a person has remitted and then relapsed in relation to GAD, any treatment received prior to remission will not be considered when deciding whether they meet criteria for TR-GAD.
4. Living in the community (i.e., domestic residences or assisted living facilities, but not care homes).

Exclusion criteria:

1. Judged to lack capacity to provide fully informed consent to participate in the trial.
2. A diagnosis of dementia or intellectual disability using standard diagnostic guidelines or clinically judged to have moderate or severe cognitive impairment.
3. A diagnosis of an imminently life-limiting illness where they would not be expected to survive the duration of the trial.
4. Expressing suicidal ideation with active suicidal behaviours/plans and active intent, as assessed using the Columbia-Suicide Severity Rating Scale Screener [23].
5. Currently receiving a course of formal psychological therapy delivered by a formally trained psychologist or psychotherapist, or unwilling to refrain from engaging in formal psychological therapy should they be randomly allocated to the ACT arm.
6. Self-report receiving ACT in the FACTOID feasibility study [19].
7. Having already been randomised in the CONTACT-GAD trial or living with another person who has already been randomised in the trial.
8. Taking part in clinical trials of other interventions for GAD.

#### Trial therapists

Inclusion criteria:

1. Aged ≥ 18 years.
2. Therapists involved in delivering the intervention within the CONTACT-GAD trial.

### Intervention

Participants will be offered up to 14 one-to-one sessions of tailored ACT, each lasting up to one hour, over six months plus a booster session at approximately 3-months post-intervention. There will be a phased ending to the sessions, such that they are approximately weekly for the first 12 sessions and then approximately fortnightly thereafter, to facilitate ending of sessions. Partners or family members will be invited to attend all sessions, with the participant’s consent. However, sessions will be focused on the participant rather than other attendees. Sessions will be delivered face-to-face (within the outpatient clinic, GP surgery or participant's home), or via video call or telephone (if video call is not available), depending on participant preference, therapist availability and service restrictions. Sessions will be delivered by Band 6–8 clinical psychologists, counselling psychologists, psychotherapists or high-intensity CBT therapists (or their equivalent in Australia) who are based in primary or secondary care services, with a minimum of one year of experience of delivering psychotherapy interventions.

As shown in Table 2, sessions will focus on the six core processes in ACT. Suggested skills, metaphors and/or experiential exercises, audio files and home practice tasks tailored to the needs and preferences of older people with TR-GAD are specified in each session. However, therapists are given the choice of what order to deliver the sessions in (based on the case conceptualisation), which ACT metaphors and/or experiential exercises to use (and personalise) in each session, and the pace of the sessions (based on individual needs and preferences). The booster session at 3-months post-intervention will review ACT skills and strategies discussed in the sessions and will be conducted after the outcome assessment at 6 months follow-up in order to avoid biasing outcomes at this timepoint.

**Table 2:** Outline of the tailored ACT intervention for older people with TR-GAD

**Session**^a^	**Main focus of the session**^b,c^	**ACT metaphors and/or exercises**
1	Assessment of current issues, goals for therapy and introduction to ACT	1) Choice point model
2–13^d^	Clarifying values (i.e., what a person wants to be doing and the way in which they want to be doing that)	1) Lifetime achievement award, Values list or Values questions
	Evaluating progress towards values (i.e., the degree to which the person is living their life in accordance with their values)	1) Pieces of the pie or Life compass
	Noticing the workability of focusing energy on 'feeling better' (i.e., trying to control, change, avoid or get rid of worry and anxiety)	1) (If time allows) Chinese finger trap exercise, Tug of war with a monster, Pushing paper exercise, Holding a book or Passengers on the bus
	Recognising the futility of focusing energy on 'feeling better' (i.e., noticing the paradox of emotional control and willingness as the alternative to control)	1) Polygraph machine 2) Willingness and anxiety dials, Chinese finger trap exercise, Tug of war with a monster, Pushing paper exercise, Holding a book or Passengers on the bus
	Developing skills for being willing to experience difficult thoughts, feelings and sensations (i.e., introducing the notion of willingness as a choice and practicing opening up to difficult internal experiences)	1) Swamp metaphor or Ticket metaphor 2) (If time allows) Observe, breathe and open up, Physicalising exercise, Accepting all of you or Cactus exercise
	Noticing the workability of a lack of contact with the present moment (i.e., getting caught up in worrying about the future or ruminating about the past)	1) Tracking thoughts in time
	Developing present moment awareness (i.e., practicing skills for staying more connected with the present moment)	1) Tracking thoughts in time, Dropping anchor exercise, Mindful eating/drinking/walking, or Observe, breathe and open up
	Noticing the workability of fusion with thoughts, images and memories (i.e., buying into or getting hooked by thoughts, images and memories) and practicing skills for defusing from unhelpful thoughts, images and memories	1) Think the opposite 2) "I'm noticing I'm having…", Imagine a thought on a computer screen, "Milk, milk, milk", Writing the thought in different colours/different styles/reverse order, or Singing or saying the thought in a silly voice
	Developing skills for defusing from unhelpful thoughts, images and memories (i.e., practicing skills for unhooking or stepping back from unhelpful thoughts, images and memories)	1) Leaves on a stream 2) (If time allows) "I'm noticing I'm having…", Imagine a thought on a computer screen, "Milk, milk, milk", Writing the thought in different colours/different styles/reverse order, or Singing or saying the thought in a silly voice
	Noticing the workability of being fused with labels or self-stories and developing skills for defusing from them (i.e., practicing skills for holding labels or self-stories lightly rather than tightly)	1) Labels exercise, House and furniture metaphor, Cup and contents metaphor, Connecting with the noticing you or Your kind friend
	Overcoming external barriers (e.g., physical health issues) using selection, optimisation and compensation principles	1) Part 1 of Doing what matters exercise, incorporating strategies for selecting or adapting goals, optimising chances of achieving goals and compensating for deficits
	Choosing and taking action to 'live better' rather than 'feel better' (i.e., identifying ways to live their life in accordance with their values, alongside worry and anxiety)	1) Part 2 of Doing what matters exercise, focusing on setting values-based goals and actions and identifying strategies for managing internal barriers (e.g., worry, anxiety)
14	Reviewing aims of ACT and key skills and concepts, positively reinforcing behavioural changes and exploring how gains can be maintained	-
Booster^e^	As above	-

^a^Sessions are approximately weekly for the first 12 weeks and then approximately fortnightly thereafter

^b^Therapists are encouraged to bring in other ACT processes throughout each session, in addition to the main focus of the session

^c^For those interested in withdrawing from or discontinuing medication, drugs and/or alcohol, the manual also includes an optional exercise focused on psychoeducation, identifying risks and benefits, and highlighting the best ways to withdraw from or discontinue medication, drugs and/or alcohol. Participants are advised to discuss any gradual withdrawal program with their psychiatrist and/or GP (or equivalent healthcare provider in Australia)

^d^Therapists are given the choice of what order to deliver the sessions in, based on the case conceptualisation, which ACT metaphors or experiential exercises to use (and personalise), and the pace of the sessions, based on individual needs and preferences

^e^Participants are offered a booster session approximately three months after the final session

Therapists will attend a 4-day experiential ACT training workshop, delivered via video call by ACT-trained members of the research team with a minimum of five years of experience in delivering ACT and training therapists to deliver ACT in clinical trials. Training will comprise a combination of didactic learning through teaching and demonstrations, experiential learning through personal experience of ACT metaphors and exercises, and practical learning through roleplays with other therapists. Training will be supplemented by a therapist manual, accompanying participant workbook and audio files and freely available online ACT resources. Training will include interested Patient and Public Involvement (PPI) representatives, where possible.

After completing training, therapists will be asked to practice delivering ACT to a service user on their caseload, under supervision, before commencing intervention delivery (assuming satisfactory competence in ACT delivery is achieved). Therapists will be invited to attend fortnightly group supervision and consultation sessions via video call, though sessions will be available on a weekly basis to make them as accessible as possible. This will be provided by ACT-trained members of the research team with a minimum of five years of experience in delivering ACT and supervising ACT within clinical trials. Therapists will also be able to receive support through a secure, supervisor-moderated online forum. Approximately 12 months after completion of the initial training, therapists will attend a 1-day top-up training course via video call to review and consolidate ACT skills.

### Comparator

Participants in both arms will receive all aspects of UC, with the exception of courses of formal psychological therapies for those randomly allocated to the ACT arm. UC will comprise standard care as outlined in NICE Clinical Guideline 113 for GAD [5]. It is likely that this will comprise: i) pharmacotherapy managed by a GP (or an equivalent healthcare provider in Australia); or ii) care by a GP (or an equivalent healthcare provider in Australia), with a multidisciplinary team within secondary care providing input in the form of assessment, psychotropic medication review and management, and case management (and psychotherapy and/or occupational therapy for a smaller proportion of participants). UC in Australia is similar to the UK and will comprise any or a combination of pharmacotherapy, supportive counselling by allied health staff and psychological therapy of various modalities.

As some variations in UC are anticipated across participants and sites, this will be monitored using a modified Client Service Receipt Inventory (CSRI) [24]. Those randomly allocated to the ACT arm will be asked to refrain from concurrent formal psychological therapies since this may lead to conflicts in therapeutic approaches and goals. No other attempts will be made to actively discourage participants from seeking treatment outside of the trial for ethical reasons. All psychological and psychotropic pharmacotherapy will be monitored and recorded throughout the course of the trial and additional exploratory data analyses examining the impact of this will be undertaken, if necessary. Sensitivity analyses will examine the consistency of outcomes across psychotropic medication use.

### Outcomes

The primary outcome measure will be the Generalised Anxiety Disorder Assessment-7 (GAD-7) [25]. This is a 7-item self-report measure of GAD, which is routinely used with adults of all ages within primary and secondary care in the NHS. The GAD-7 will be completed at baseline (0 months), following confirmation of eligibility and consent, 6 months post-randomisation (the primary endpoint), and 12 months post-randomisation.

Secondary outcome measures will be completed at the same time points, unless otherwise stated, and will include:

- McGill Quality of Life Questionnaire-Revised [26]: This is a self-report measure of quality of life that has good psychometric properties. It comprises 14 items forming 4 subscales: Physical (3 items), Psychological (4 items), Existential (4 items) and Social (3 items);
- Geriatric Depression Scale-15 [27]: A 15-item self-report measure of depression developed specifically for older people;
- Comprehensive Assessment of ACT processes (CompACT) [28]: A 23-item self-report measure of psychological flexibility, which ACT aims to develop. It has 3 subscales: openness to experience (which explores one’s willingness to experience thoughts, emotions, sensations, etc.), behavioural awareness (which assesses mindful awareness of one’s actions), and valued action (which examines engagement in meaningful activities);
- Health and social care resource use, including dose and frequency of prescribed and non-prescribed medication: This will be captured using a modified CSRI [24];
- EQ-5D-5L plus EQ-VAS [29]: A 5-item self-report measure and visual analogue scale measure of health-related quality of life. The former will be used to calculate utility scores for quality-adjusted life years;
- ICECAP-O [30]: A 5-item self-report capability measure for older people, which captures benefits to broader wellbeing than just health and will be used to calculate capability-adjusted life years;
- Adverse events (e.g. falls, new reports of suicidal ideation, deaths, hospitalisations, etc.) at 6- and 12-months follow-up;
- Satisfaction with ACT plus UC or UC alone: This will be assessed using the Client Satisfaction Questionnaire-8 [31] and will be assessed in both arms at 6-months follow-up in order to avoid unblinding of outcome assessors;
- Goal-Based Outcomes tool [32]: A self-reported, idiographic outcome measure will be used to assess personally meaningful behaviour change. This asks a person to define three personally meaningful behavioural goals and then rate their progress towards this goal on an 11-point Likert scale (from 0 = not met at all to 10 = fully met);
- Cognitive & Leisure Activity Scale [33]: A 16-item self-report measure that assesses engagement in 16 types of activities, including cognitive, social, creative and spiritual activities;
- Adherence (i.e., session attendance after each session for those randomly allocated to the ACT arm).

### Measures of bias

Measures of bias will include:

- Expectations about treatment: Prior to randomisation, older people with TR-GAD will be asked to rate how much they expect their symptoms to improve and how much they expect their life to improve if they receive ACT on a 5-point Likert scale from 0 (not at all) to 4 (completely). Therapists will be asked to rate the same questions after a participant's first therapy session;
- Treatment preferences: Prior to randomisation, older people with TR-GAD will be asked to rate how much they would hope to receive ACT plus UC and how much they would hope to receive UC alone on a 5-point scale from 0 (not at all) to 4 (completely);
- Contamination in the control arm: Receipt of other forms of psychological and pharmacological treatment for GAD outside of the trial will be recorded using the modified CSRI. Additional exploratory data analysis will be undertaken if reported by a substantial proportion of participants;
- *A*ssessment of blindness of outcome assessors: Outcome assessors will be asked to declare if they have been unblinded (and how) at 6- and 12-months follow-up. Those who have not been unblinded will be asked to guess whether they think participants were allocated to the intervention or control arm.

### Treatment fidelity

Treatment fidelity will be assessed in four areas:

- Training: Training workshops will be videoed and an independent ACT therapist will assess the fidelity of training to the ACT model. Therapists' knowledge of ACT will be assessed through their responses to a clinical vignette-based exercise at the end of training;
- Treatment delivery: All therapy sessions will be audio-recorded using an encrypted digital voice recorder, and 10% of randomly selected sessions will be rated on an ongoing basis throughout intervention delivery by an independent, experienced ACT therapist using the ACT Fidelity Measure [34]. The ACT-FM is a 25-item measure, which assesses ACT fidelity in 4 domains (open response style, aware response style, engaged response style and therapist stance). Scores for each subscale are summed in order to produce a total ACT consistency score and a total ACT inconsistency score. In addition, adherence to the treatment manual and therapy components will be measured using a checklist that therapists complete at the end of each session, which will be adapted from previous work [19];
- Treatment receipt: The Comprehensive Assessment of ACT processes [28] will be used to measure changes in psychological flexibility in older people with TR-GAD. Engagement with therapy will be defined by the number of sessions out of 14 attended: poor (0–3), moderate (4–6), good (7–10), excellent (11–14);
- Treatment enactment: An idiographic patient-reported outcome measure, the Goal-Based Outcomes tool [32], will be used to assess personally meaningful behavioural changes.

### Participant timeline

**Fig. 1:**
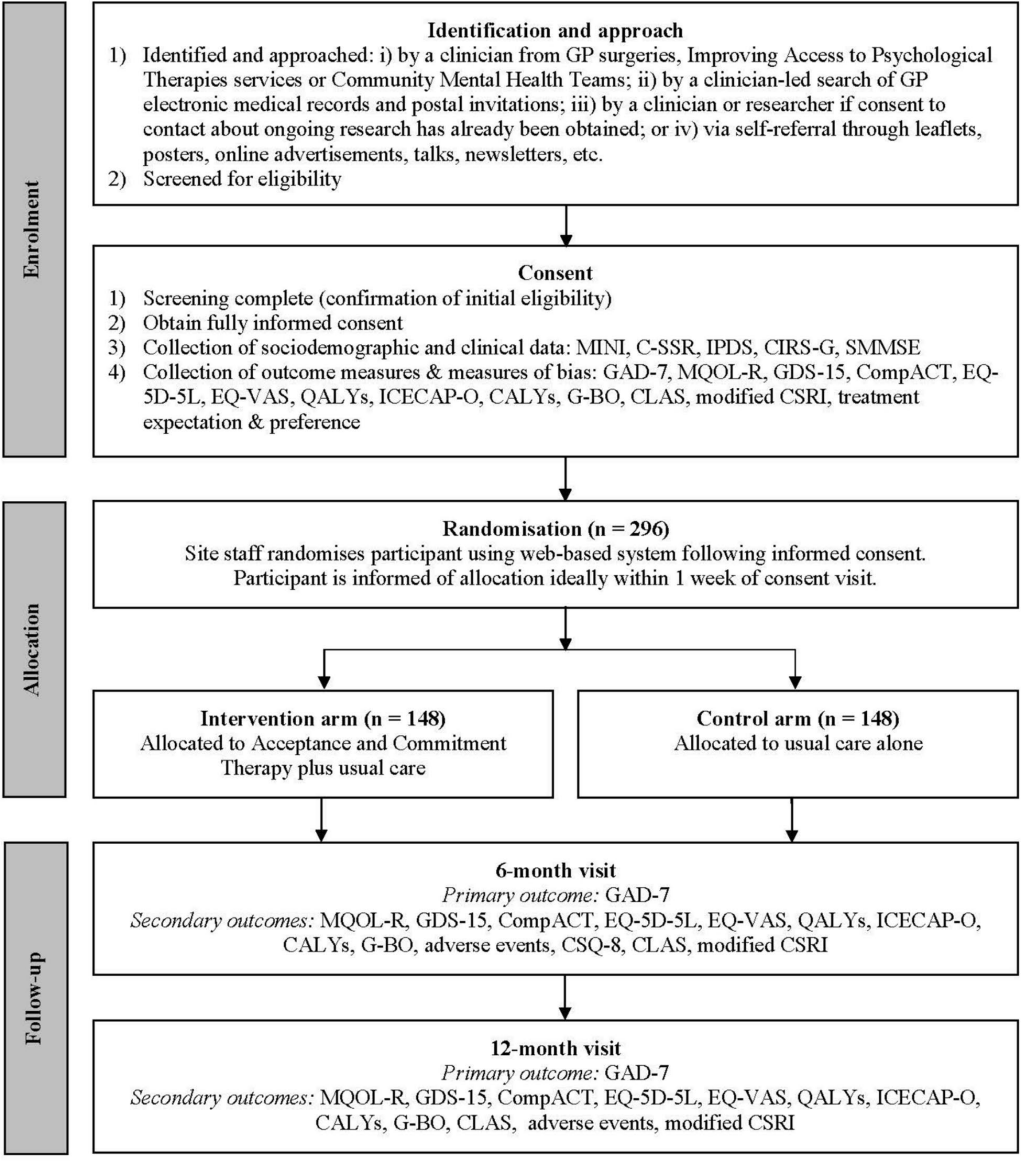
Timeline for older people with TR-GAD in the trial Notes: C-SSR = Columbia-Suicide Severity Rating Scale Screener, CALYs = Capability-adjusted life years, CIRS-G = Cumulative Illness Rating Scale-Geriatrics, CLAS = Cognitive & Leisure Activity Scale, CompACT = Comprehensive Assessment of ACT processes, CSQ-8 = Client Satisfaction Questionnaire-8, CSRI = Client Service Receipt Inventory, EQ-5D-5L = EuroQol-5 domains-5 levels, EQ-VAS = EuroQol visual analogue scale, G-BO = Goal-Based Outcomes tool, GAD-7 = Generalised Anxiety Disorder Assessment-7, GDS-15 = Geriatric Depression Scale-15, ICECAP-O = ICEpop capability measure for older people, IPDS = Iowa Personality Disorder Screen, MINI = Mini-International Neuropsychiatric Interview, MQOL-R = McGill Quality of Life Questionnaire-Revised, QALYs = Quality-adjusted life years, SMMSE = Standardised Mini-Mental State Examination

As shown in Fig. 1, older people with TR-GAD will be involved in the RCT for approximately 12 months (± 6 weeks) after randomisation.

### Sample size

296 older people with TR-GAD (148 per arm) will be recruited from approximately 15 sites (11 in the UK and 4 in Australia). This will allow detection of an effect size of 0.37 standard deviations (SD), with a two-sided alpha of 5% and 90% power. This assumes: a) a correlation of 0.55 between scores at 0- and 6-months, as seen in our previous feasibility study [19]; b) 20% attrition at 6-months [19]; and c) an intraclass correlation coefficient of 5% among 30 therapists (two per site) in the intervention arm, similar to previous studies [35]. In order to maintain a 1:1 allocation per arm, the sample size will be modified to 148 participants per arm, which is sufficient to maintain 90% power.

Our effect size of 0.37 SD is based on the fact that: a) improvements of 3–4 GAD-7 units are regarded as clinically important changes to individual patients [36–39]; and b) a change of approximately 2 GAD-7 units (0.4 SD) would mean an additional 15% of people having a clinically important improvement of 3 units compared with UC, based on Normal distributional theory. This is similar to the 0.40–0.46 SD difference observed in systematic reviews of ACT for mental and physical health conditions and CBT for GAD [40–42]. Our effect size has been reduced from 0.4 to 0.37 SD in order to compensate for the inclusion of people with limited or no spoken English necessitating the use of an interpreter, which may affect engagement with ACT.

### Recruitment

#### Older people with TR-GAD

Potentially eligible participants will be identified and approached about the trial through one of four routes: a) local clinicians or clinical team administrators from GP surgeries, NHS Talking Therapies services and Community Mental Health Teams (or their equivalent in Australia); b) searches of GP electronic medical records (or their equivalent in Australia) and postal invitations to identified potentially eligible participants; c) self-referral through community and online advertisements; and d) clinical databases (in which people have already given consent for research contact) and research databases (including Join Dementia Research and the NIHR Be Part of Research Volunteer Service in the UK).

Many older people who meet diagnostic criteria for GAD are referred to primary and secondary care services with a diagnosis of major depression and comorbid anxiety or mixed anxiety and depression rather than GAD. Consequently, clinicians in the services noted above or a member of the local or central research team will pre-screen potential participants who are referred with these diagnoses (rather than GAD) using the Generalized Anxiety Disorder-2 (GAD-2), if they provide verbal consent to this. The GAD-2 is a 2-item questionnaire used to identify GAD in primary care [43]. If a potential participant scores ≥ 2 points on this scale [44], they will be asked to complete the Patient Health Questionnaire-2 (PHQ-2). This is a 2-item questionnaire used to identify depression in primary care [45]. If the PHQ-2 total score is higher than the GAD-2 total score then they will be asked whether the symptoms of depression or GAD are most distressing, severe or of most concern to them. If symptoms of GAD are most distressing, severe or of most concern to them, or if symptoms of GAD and depression are equally problematic, then the study will be further discussed with them.

Once potentially eligible participants have been identified and verbal consent for contact has been obtained, a member of the local or central research team will discuss the trial with them, either in person or via video call, telephone or email. If they express an interest in participating in the trial, they will be asked to verbally consent to completing the GAD-2 screening questionnaire, if not already completed. If they score ≥ 2 points on the GAD-2 and they continue to express an interest in participating in the trial then they will be given a Participant Information Sheet. If they are still interested in participating in the trial, the member of the local or central research team will arrange a screening appointment, either in person or via telephone or video call. During this appointment, fully informed written consent, audio-recorded verbal consent (via telephone or video call) or digital consent (via email or Qualtrics) to take part in the trial will be sought. Following this, eligibility for inclusion in the study will be determined through a screening interview.

Those who speak English as a second language or who speak no English necessitating the use of an interpreter will not be excluded. However, they will complete study procedures and outcome measures through interpreters, where necessary. Participant-facing documents such as the Participant Information Sheet, consent form, recruitment leaflet and recruitment poster will be translated into languages other than English where possible.

#### Trial therapists

Participants will be recruited from the group of trial therapists who will be involved in delivering the intervention to older people with TR-GAD. They will be approached about completing a qualitative satisfaction questionnaire by a member of the central research team. Other procedures will be similar to those described above.

### Randomisation

Eligible participants with TR-GAD will be randomised in a 1:1 ratio to one of two arms (ACT plus UC or UC alone) using a web-based, centralised randomisation system hosted by the Sheffield Clinical Trials Research Unit (SCTRU). Randomisation will be stratified by recruitment site. The concealed allocation sequence will be hosted by the SCTRU in accordance with their standard operating procedures (SOPs) and will be held on a secure server. Access to the concealed allocation sequence will be restricted to those with authorisation. A SCTRU statistician will set up the randomisation system, but neither statistician nor other trial team members will be able to view the randomisation list during the trial. Eligible participants will be randomised once fully informed consent has been provided and baseline measures have been collected.

### Blinding

At least one trial statistician will be blinded to allocation during the trial. It is intended that the outcome assessor will be blind to treatment allocation for the duration of the trial, while older people with TR-GAD, trial therapists and clinicians will be aware of this. Only the Data Monitoring and Ethics Committee (DMEC) will have access to unblinded data at their request during the trial. Any instances of accidental unblinding will be recorded at 6- and 12-months follow-up.

### Data collection

Fully informed consent will be obtained from all participants prior to any data collection. For older people with TR-GAD, data pertaining to socio-demographic and clinical characteristics will be collected at screening and baseline (see Fig. 1). Data collection will be conducted in person (at home or in clinic) or via video call, telephone, online via Qualtrics or post at 0 months, 6 months post-randomisation (± 6 weeks) and 12 months post-randomisation (± 6 weeks) by a blind outcome assessor. Table 3 lists exceptions to this. Mode of administration will be recorded at each time point. Numerous strategies will be used to promote participant retention, including the provision of non-contingent vouchers for completion of outcome measures at follow-up.

**Table 3:**
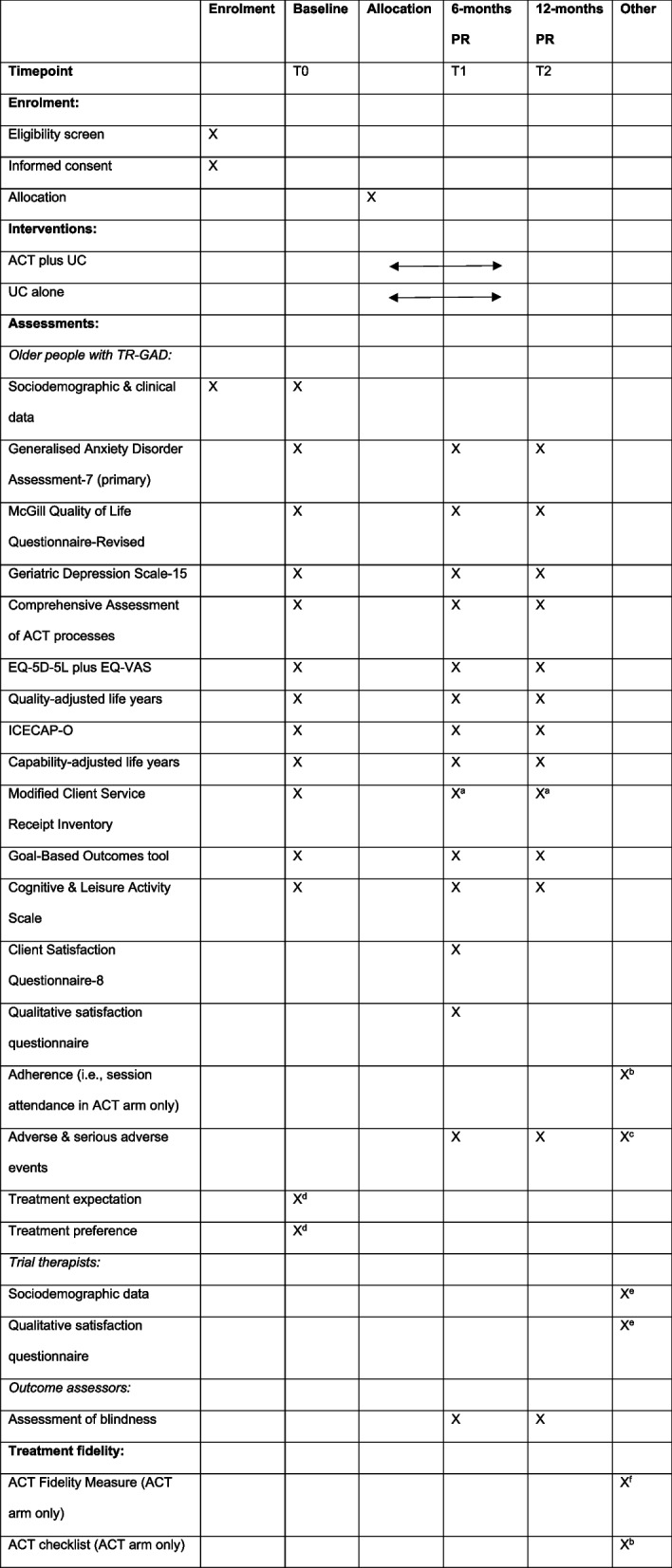
Schedule of enrolment, interventions and assessments

*ACT* Acceptance and Commitment Therapy, *PR* post-randomisation, *UC* usual care

^a^As the modified CSRI includes a question about psychological therapies received, this will be administered in one of four ways at follow-up to prevent potential unblinding of outcome assessors: i) returned via post to the central study team; ii) via online methods; iii) by telephone by the non-blind outcome assessor arranging the follow-up visit, with the rest of the assessment being completed by the blinded outcome assessor; or iv) at the end of the outcome assessment session at 12 months, after the outcome assessor has completed the unblinding question

^b^After each session

^c^Serious adverse events can be reported at any time

^d^Completed after consent, but prior to randomisation, after participants are given a rationale for ACT

^e^Completed at the end of involvement in the trial

^f^Assessed on an ongoing basis throughout intervention delivery in 10% of randomly selected sessions

All older people with TR-GAD will be invited to complete an anonymous qualitative satisfaction questionnaire at 6-month follow-up via post, email or online via Qualtrics (or verbally via telephone, video call or face-to-face interview if necessary). Any questionnaires completed verbally will be conducted by an independent member of the local or central research team to avoid unblinding of outcome assessors. There will also be separate versions of the questionnaire for the intervention arm and UC arm to avoid unblinding of outcome assessors. Those in the intervention arm will be asked questions in relation to the acceptability of ACT and its suitability and relevance to older people with TR-GAD, perceived benefits and limitations of the intervention, perceived mechanisms of impact, facilitators of and barriers to implementing the intervention in their everyday lives, and recommendations for revising the intervention. Those in the UC arm will be asked questions in relation to the psychological aspects of their usual care. Questions will focus on what kind of formal and informal psychological support they received (if any), what was helpful and what was not, and what they felt would have been helpful.

All trial therapists will also be invited to complete an anonymous qualitative satisfaction questionnaire at the end of delivering ACT in the trial. This will collect brief data on socio-demographic and professional characteristics. It will then ask a combination of closed and open questions in relation to how ACT was delivered in practice, facilitators of and barriers to implementing the intervention in the NHS, and recommendations for revising the intervention.

### Data management

Study-specific procedures for data management will be detailed in a data management plan. Data collection, management and analysis will be overseen by SCTRU, who will ensure that the trial is undertaken according to SCTRU SOPs and Good Clinical Practice guidelines. Data will be collected and retained in accordance with the UK's Data Protection Act (2018), which complies with the Australian Privacy Principles (APP) set out in the Australian Privacy Act (1988).

Participants will be assigned unique identification codes, which will be used in all data storage and will not contain any names or other personally identifiable information. Case report forms will not bear the participant’s name or other personal identifiable data. Any personally identifiable information (such as contact details) will be stored in locked cabinets. No identifiable Australian patient data will be shared with the UK team. Confidentiality will be kept unless there is evidence of risk of harm to self or others.

Qualtrics will be used as a digital option to collect informed consent and trial data. Qualtrics has obtained ISO 27001, ISO/IEC 27017, ISO/IEC 27018 and ISO 9001 security certifications, which are internationally recognised, best practice frameworks for information security management systems. The SCTRU’s web-based data management system, Prospect, will be used to store trial data in a PostgreSQL database on virtual servers hosted by Corporate Information and Computing Services at the University of Sheffield. Prospect uses industry standard techniques to provide data security, including password authentication and encryption using Secure Sockets Layer/Transport Layer Security. Australian participants will be asked to consent to their personal and research data being transferred to and stored by the University of Sheffield within Prospect.

Verbal consent for trial participation, audio content of therapy sessions and verbal responses to qualitative satisfaction questionnaires (for those unable to complete a written version of this) will be audio recorded using encrypted digital voice recorders or Microsoft Teams recording functionality. Audio files will be uploaded to a secure server using University College London’s Data Safe Haven, which satisfies the highest level of security requirements of NHS trusts. They will then be transferred and stored on UCL’s password protected secure electronic network. Australian participants will be asked to consent to their audio files being transferred to and stored by University College London’s Data Safe Haven.

In line with the sponsor’s data protection policy, UK study documentation and pseudonymised data will be securely kept for a period of 10 years following completion of the study. Australian study documentation and pseudonymised data stored in Australia will be securely kept for a period of 15 years following completion of the study.

### Statistical methods

A statistical analysis plan will be developed, reviewed and approved by the Trial Steering Committee (TSC) prior to data analysis. The primary outcome will be analysed using multi-level modelling, which will include fixed effect covariates (treatment arm and baseline score) and a random effect covariate (therapist) to account for potential clustering. Separate analyses will be conducted at 6-months (primary analysis timepoint) and 12-months follow-up. The difference between treatment arms in mean GAD-7 total score and its 95% confidence interval will be quantified by the model coefficient. Primary analyses will be by intention to treat, but additional sensitivity analyses will assess the impact of session uptake using complier-average causal effect (CACE) analyses to model the average treatment effect among those who were considered “compliant” with ACT. For the purpose of trial data analysis, completion of seven sessions will be regarded as a minimum number allowable for an adequate exposure to treatment in the protocol, with participants that receive fewer than seven sessions being a deviation from this. As the minimum dose can vary across participants, this will be assessed further using a CACE analysis in which treatment outcome will be examined in relation to the number of sessions received. In addition, sensitivity analyses will examine the consistency of outcomes across sites, baseline GAD severity, age at first onset and baseline psychotropic medication use.

Secondary outcomes will be analysed in a similar manner to the primary outcome. Additional exploratory analyses will be undertaken to assess the consistency of treatment effects across a variety of subgroups. These will include treatment preference and expectations, psychiatric comorbidity, limited or no spoken English skills, country of recruitment and mode of therapy delivery. The impact of contamination (e.g. psychological therapy in the control arm) will be assessed in a per-protocol analysis [46].

It is expected that there will be missing outcome data for some participants, either due to study withdrawal, loss to follow up or death. The number of missing values will be summarised by treatment arm, time point and reason. Multiple imputation using Rubin's rules [47] will be implemented for the primary endpoint. Adverse events will be summarised in terms of the number and percentage of participants experiencing each event and the number of events by treatment arm.

### Economic evaluation

A health economic analysis plan will be developed, reviewed and approved by the TSC prior to data analysis. A within-trial cost-utility analysis will present the incremental costs per quality-adjusted life year gained of older people with TR-GAD receiving tailored ACT plus UC compared to UC alone from an NHS and social care perspective. Costs will be estimated on a per-participant basis and will include costs for delivering the intervention. The modified CSRI will be used to collect data on health and social care resource use. Unit costs will be derived from relevant national sources and will include NHS reference costs and Personal Social Service Research Unit costs [48]. The standard version of the EQ-5D-5L will be used to collect patient reported health status. Values for EQ-5D-5L for England will be used based on NICE advice at the time of analysis, which may either be to use the value set currently in collection or a mapping approach. These will be calculated using the area under the curve method. Appropriate multiple imputation techniques will be implemented where data on the EQ-5D-5L or resource use are missing. Differences in costs and quality-adjusted life years between the treatment arms will be described and the incremental cost effectiveness ratio, with associated uncertainty, will be calculated.

Clinical effectiveness data will be used to judge whether there is evidence of continued benefit from the treatment at 12 months and any evidence of a waning of effect. This will determine if there are grounds to extrapolate the analysis beyond the 12 months observed period using a simple decision model to estimate costs and benefits. This may be important since continued health benefits are unlikely to be matched by increased costs, given the upfront costs of providing the intervention. The time period for the model or appropriate methods for extrapolation cannot be determined at this stage. Any model based extrapolation will adhere to standard methods to reflect uncertainty including probabilistic sensitivity analysis and one-way/multi-way analyses. A separate analysis of over-the-counter medication will also be conducted in order to assess whether there are significant differences between treatment arms. A sensitivity analysis including these costs will be conducted if differences are non-negligible. Similar analyses will be conducted for capability-adjusted life years from the ICECAP-O.

With respect to the pooling of UK and Australian data, the base case analysis will pool data on both outcomes and resource use from all participating sites in the UK and Australia as usual care and health systems are considered to be similar in both countries and resource use is expected to be comparable. UK-specific unit costs and UK/England EQ-5D index scores will be applied to the participant level data and the analysis will proceed on the full dataset, maximising use of the trial data. Multilevel modelling of costs and outcomes will be used in a sensitivity analysis, to explore the potential impact of clustering at the national and/or therapist level. An exploratory analysis of treatment effect will be conducted by country of recruitment.

### Mixed-methods process analysis

An informal mixed-methods process analysis will be conducted to examine perceived mechanisms of impact, facilitators of and barriers to implementation, and contextual factors. Qualitative data from the qualitative satisfaction questionnaire, completed by older people with TR-GAD at 6-months follow-up and trial therapists at the end of their involvement in the study, will be transcribed verbatim and anonymised to maintain confidentiality. Data will be analysed iteratively using focussed thematic analysis [49, 50]. Two members of the research team will independently code initial questionnaires using the computer programme, NVivo, before constructing an analytical framework around: i) the acceptability, suitability, relevance, perceived benefits and limitations, perceived mechanisms of impact, and facilitators of and barriers to implementation of ACT for older people with TR-GAD for those in the intervention arm; and ii) the psychological support received, what was felt was needed, and the helpfulness of psychological support for those in the usual care arm. The analytical framework will be applied to the remaining questionnaires, with themes and subthemes being refined as necessary. Ideas about themes and relationships will be discussed with PPI representatives. Findings will be used to make further refinements to the intervention, particularly with respect to implementation in clinical practice.

Quantitative data relevant to the process analysis will focus on four key areas: intervention uptake, treatment fidelity, reach and outcomes. Data collected on number of sessions attended, modality of sessions, use of interpreters and reasons for non-attendance will be analysed to explore what contextual factors (such as participant sociodemographic and clinical characteristics at baseline) may influence uptake of the intervention. Data collected on ACT consistency and inconsistency scores from the ACT Fidelity Measure will be analysed to explore what contextual factors (such as therapist characteristics at baseline and mode of delivery) may influence treatment fidelity. Sociodemographic data from the trial will be analysed to explore reach and uptake in eligible populations in diverse settings and identify any under-represented populations through comparison with Office of National Statistics area level census data. Sensitivity analyses and additional exploratory analyses will identify what contextual factors (such as clinical characteristics at baseline) are associated with variations in primary and secondary outcome data.

### Trial oversight

The study will be conducted in line with the Helsinki Declaration. North London NHS Foundation Trust (formerly Camden and Islington NHS Foundation Trust) is the nominated sponsor and will lead research governance. The study will be conducted in accordance with the protocol, SCTRU SOPs and Good Clinical Practice. Three committees will govern the conduct of the trial: the TMG, TSC and DMEC. The TMG will comprise co-applicants, collaborators, PPI representatives, and trial staff. It will initially meet monthly via video call and then every 2–3 months as the trial progresses. The independent TSC will comprise academic clinicians, a statistician, a health economist and PPI representatives, while the independent DMEC will comprise academic clinicians and a statistician. Both groups will meet every 6–12 months to review progress and monitor the trial, with safety data additionally being reviewed by the DMEC.

### Safety

Adverse Events (AEs) and Serious Adverse Events (SAEs) can be reported by trial sites at any stage of trial participation, including by participants at 6- and 12-months follow-up, in accordance with SCTRU SOPs. An AE will be defined as any untoward medical occurrence in a trial participant with TR-GAD. Categories of AEs and SAEs are shown in Table 4. All SAEs will be reported to the SCTRU and the sponsor within 24 h of discovery at the trial site. SAEs will be rated in terms of seriousness, intensity, frequency, relationship to the intervention and expectedness. Those deemed both “unexpected” and “related” to the intervention will be reported to the REC within 15 days of being reported to the trial team. In addition, the Australian research team will report SAEs for participants recruited from Australian sites to their Research Governance Office within 72-h, in line with National Health and Medical Research Council requirements. Compensation to UK and Australian participants who suffer harm from participation in the trial will be available through insurance held by North London NHS Foundation Trust and Macquarie University, respectively.

**Table 4:** Definition of adverse events (AEs) and serious adverse events (SAEs) in the trial

Type of event	Categories
AE	Any new co-morbid psychiatric condition reported
	Any reported event that has significantly affected the psychological health status of the participant (e.g. a stressful life event such as a bereavement)
	New reports of suicidal ideation with or without active suicidal behaviour/plans, but without intent during the trial (i.e. not reported at baseline)
	Other
SAE^a^	New reports of suicidal ideation with active suicidal behaviour/plans and intent
	Reports of physical self-harm
	Requires unplanned in-patient hospitalisation^b^
	Requires prolongation of existing hospitalisation^b^
	Is life-threatening^c^
	Results in persistent or significant disability or incapacity
	Results in death
	Considered medically significant by the investigator

^a^All of the SAEs defined here will be classified as unexpected

^b^Hospitalisation is defined as an inpatient admission, regardless of length of stay, even if the hospitalisation is a precautionary measure for continued observation

^c^A ‘life-threatening’ event refers to an event in which the participant was actually at risk of death at the time of the event

### Ethics

The trial has been approved by the West of Scotland Research Ethics Committee and Health Research Authority (22/WS/0186) in the UK and the Human Research Ethics Committee in Australia (520,231,567,953,925). Any amendments to the trial protocol will be approved by the sponsor and communicated to the Health Research Authority and all sites. Recruitment will only commence at a site when: a) written confirmation of capability and capacity (or equivalent organisation approval in Australia) has been provided by the site, b) the site has completed a Site Initiation Visit; and c) the sponsor (or its delegated representative) has issued the green light to commence recruitment at the site.

Older people with TR-GAD and trial therapists will be consented in line with the Mental Capacity Act (2005) and SCTRU SOPs. All participants will be asked to provide fully informed written consent, audio-recorded verbal consent (if being obtained by telephone or video call) or digital consent (via email or an online consent form via Qualtrics) to take part in the trial. No trial procedures will be conducted prior to participants giving consent to participate in the trial. Participants will be made aware that participation is voluntary and they may withdraw from the intervention and/or the trial at any time, without having to give a reason and without it affecting their care or legal rights. They will also be made aware that they may be withdrawn from the trial if participation is no longer in their best interests. Participants will be made aware that if they choose to withdraw from the trial and not complete further follow-up assessments, any data already provided by them will remain in the full dataset for intent-to-treat analysis.

### Patient and public involvement

Older people with lived experience of TR-GAD were involved in our previous FACTOID feasibility study and in the design of the CONTACT-GAD trial. They will continue to be involved in the trial in numerous ways. A PPI group comprising approximately 6–7 older people with lived experience of GAD will meet approximately every 6 months in the first 2 years of the study and annually thereafter via video call. They will discuss a range of issues, including study progress, recruitment strategies, study materials, and interpretation and dissemination of findings. Interested PPI representatives will also be invited to engage in a range of other activities, including: a) attending Trial Management Group (TMG) and Trial Steering Committee (TSC) meetings; b) participating in training of therapists from a lived experience perspective; c) participating in presentations about key findings; and d) co-writing articles about key findings for a public audience.

### Dissemination

Dissemination to the academic and clinical community, service users and the broader public will occur through: a) peer-reviewed, international, open-access academic journals (standard author eligibility guidelines will be followed); b) blogs about key findings co-written with PPI representatives and a summary of the research findings for interested trial participants; c) academic conferences and local clinical conferences and meetings; d) talks to local service user groups; e) social media (e.g., University media releases and University website); f) ACT training and seminars; and g) the ISRCTN database.

## Conclusion

Clear evidence-based guidance regarding the management of TR-GAD in older people is lacking. This RCT will address this evidence gap by assessing the clinical and cost effectiveness of tailored ACT plus UC compared to UC alone for reducing anxiety in older people with TR-GAD. To our knowledge, this will be the first RCT to evaluate a form of psychological therapy for older people with TR-GAD. It will also be the first RCT to examine ACT, tailored to the specific needs and preferences of older people with TR-GAD, in this population.

Although findings from this RCT will potentially provide much needed guidance to the NHS regarding the management of TR-GAD in older people, there are a number of limitations. The main limitation relates to the choice of control arm. On the one hand, the use of UC as the comparator will enable ACT to be compared to what is currently available within the NHS. However, on the other hand, the use of a non-active rather than active control means that it will not be possible to determine whether any beneficial effects are due to non-specific therapeutic factors such as the provision of social support or other factors such as expectancy. Evidence that changes in psychological flexibility mediate treatment response at 6- and 12-months follow-up will help support the notion that any beneficial effects are due to the intervention itself. However, the use of a talking placebo control, such as that used in a previous RCT of Cognitive Behavioural Therapy for older people with depression [51], would have enabled us to more clearly determine this. A related limitation is the fact that it will not be possible to maintain double-blinding given that older people with TR-GAD will not be blinded to treatment arm allocation. This means that blinded outcome assessors may be inadvertently unblinded during outcome assessments at follow-up. Study procedures are in place to minimise this risk as much as possible, but it may still bias results. Consequently, this will be monitored and taken into account in statistical analyses, if necessary. A final limitation is that outcome measures will be collected at baseline and 6- and 12-months follow-up. Although this will help to inform us of the maintenance of treatment effects beyond intervention delivery, it does mean that it will not be possible to examine longer-term maintenance.

In conclusion, GAD is the most common anxiety disorder in older people. While guidance exists for the management of GAD, less is known about the management of GAD that does not respond to current first-line treatments, particularly in older people. We previously showed that a form of psychological therapy, ACT, was both feasible to deliver and acceptable to older people with TR-GAD in an uncontrolled feasibility study. We also showed that it may help to reduce anxiety in this population. However, whether these benefits were specifically due to ACT and whether this type of intervention is clinically and cost effective is unknown. This RCT aims to address these uncertainties and, despite the limitations noted above, provide crucial evidence-based guidance on the management of TR-GAD in older people.

## Supplementary Information

Supplementary Material 1: SPIRIT 2025 checklist.

Supplementary Material 2: Template for intervention description and replication (TIDieR) checklist.

Supplementary Material 3: WHO Trial Registration Data Set.

## Data Availability

No datasets were generated or analysed during the current study.

## Abbreviations

ACT: Acceptance and Commitment Therapy
AE: Adverse event
CBT: Cognitive Behavioural Therapy
CSRI: Client Service Receipt Inventory
DMEC: Data Monitoring and Ethics Committee
GAD: Generalised anxiety disorder
NIHR: National Institute for Health and Care Research
PPI: Patient and public involvement
RCT: Randomised controlled trial
SAE: Serious adverse event
SCTRU: Sheffield Clinical Trials Research Unit
SOP: Standard operating procedure
TMG: Trial Management Group
TR-GAD: Treatment-resistant GAD
TSC: Trial Steering Committee
UC: Usual care
