# Supplementary material for: A randomised controlled trial of Acceptance and Commitment Therapy plus usual care in comparison to usual care alone for reducing anxiety in older people with treatment-resistant generalised anxiety disorder (CONTACT-GAD): trial protocol
Source: BMC Geriatr. 2026 Feb 11;26:359. doi: 10.1186/s12877-026-07094-6 (PMC12998016; doi:10.1186/s12877-026-07094-6)
Supplement: Supplementary file 3 — Supplementary Material 3: WHO Trial Registration Data Set. [file 12877_2026_7094_MOESM3_ESM.docx]

## Supplementary File 3: WHO Trial Registration Data Set.

| **Data category** | **Information** |
| --- | --- |
| Primary registry and trial identifying number | ISRCTN Registry, ISRCTN85462326, <https://www.isrctn.com/ISRCTN85462326> |
| Date of registration in primary registry | 04 January 2023 |
| Secondary identifying numbers | IRAS 320523, REC 22/WS/0186, HREC 520231567953925, NIHR134141 |
| Source(s) of monetary or material support | 1. National Institute for Health and Care Research (NIHR) Health Technology Assessment Programme (NIHR134141)  2. National Health and Medical Research Council-NIHR Collaborative Research Grant Scheme (2014745) |
| Primary sponsor | North London NHS Foundation Trust (formerly Camden and Islington NHS Foundation Trust) |
| Secondary sponsor(s) | N/A |
| Contact for public queries | Rebecca Gould (r.gould@ucl.ac.uk) |
| Contact for scientific queries | Rebecca Gould (r.gould@ucl.ac.uk) |
| Public title | Acceptance and commitment therapy for older people with treatment resistant generalised anxiety disorder (CONTACT-GAD) |
| Scientific title | A randomised CONtrolled trial of Tailored Acceptance and Commitment Therapy for older people with treatment resistant Generalised Anxiety Disorder (CONTACT-GAD) |
| Countries of recruitment | UK and Australia |
| Health condition(s) or problem(s) studied | Generalised anxiety disorder |
| Intervention(s) | Acceptance and Commitment Therapy plus usual care vs. usual care alone |
| Key inclusion and exclusion criteria | *Older people:*  Inclusion criteria:   1. Aged ≥60 years. 2. Diagnosis of generalised anxiety disorder (GAD) using the Mini-International Neuropsychiatric Interview. 3. GAD that is ‘treatment resistant’, defined as GAD that has failed to respond adequately to pharmacotherapy and/or psychotherapy treatment, as described in step 3 of the UK's stepped care model for GAD. Those who have been offered pharmacotherapy and/or psychotherapy treatment and did not want to start it or continue it and are still symptomatic will also be included in this definition. An equivalent definition will be used in Australia.   Exclusion criteria:   1. Lacking capacity to provide fully informed written consent to participate in the trial. 2. Diagnosis of dementia or intellectual disability using standard diagnostic guidelines, or clinically judged to have moderate or severe cognitive impairment. 3. Diagnosis of an imminently life-limiting illness where they would not be expected to survive for the duration of the trial. 4. Expressing suicidal ideation with active suicidal behaviours/plans and active intent. 5. Currently receiving a course of formal psychological therapy delivered by a formally trained psychologist or psychotherapist, or those who are unwilling to refrain from engaging in such formal psychological therapy during the receipt of ACT. 6. Self-report having received ACT in the FACTOID feasibility study. 7. Having already been randomised in the CONTACT-GAD trial or living with another person who has already been randomised in the CONTACT-GAD trial. 8. Taking part in clinical trials of other interventions for GAD.   *Therapists:*  Inclusion criteria:   1. Aged ≥18 years. 2. Trial therapists who are involved in delivering the intervention in the trial. |
| Study type | Multi-centre, assessor-blind, parallel, two-arm randomised controlled trial |
| Date of first enrolment | 28/06/2023 |
| Sample size | 296 |
| Recruitment status | Recruiting |
| Primary outcome(s) | Generalised Anxiety Disorder Assessment-7 |
| Key secondary outcomes | - McGill Quality of Life Questionnaire-Revised - Geriatric Depression Scale-15 - Comprehensive Assessment of ACT processes - Health and social care resource use using modified Client Service Receipt Inventory - EQ-5D-5L plus EQ-VAS - ICECAP-O - Quality-adjusted life years and capability-adjusted life years - Adverse events - Client Satisfaction Questionnaire-8 - Goal-Based Outcomes tool - Cognitive & Leisure Activity Scale - Adherence (i.e., session attendance for those in the ACT arm) |
| Ethics review | Status: Approved  Date of approval: 20/12/2022  Ethics Committee: West of Scotland Research Ethics Committee and Health Research Authority in the UK and the Human Research Ethics Committee in Australia |
